# Implicit 3D Human Reconstruction Guided by Parametric Models and Normal Maps

**DOI:** 10.3390/jimaging10060133

**Published:** 2024-05-29

**Authors:** Yong Ren, Mingquan Zhou, Yifan Wang, Long Feng, Qiuquan Zhu, Kang Li, Guohua Geng

**Affiliations:** 1 School of Information Science and Technology, Northwest University, Xi’an 710127, China; ryrory@stumail.nwu.edu.cn (Y.R.); 202221524@stumail.nwu.edu.cn (Y.W.); fenglong@stumail.nwu.edu.cn (L.F.); zhuqiuquan@stumail.nwu.edu.cn (Q.Z.); ghgeng@nwu.edu.cn (G.G.); 2 National and Local Joint Engineering Research Center for Cultural Heritage Digitization, Xi’an 710127, China

**Keywords:** human reconstruction, parametric model, local feature

## Abstract

Accurate and robust 3D human modeling from a single image presents significant challenges. Existing methods have shown potential, but they often fail to generate reconstructions that match the level of detail in the input image. These methods particularly struggle with loose clothing. They typically employ parameterized human models to constrain the reconstruction process, ensuring the results do not deviate too far from the model and produce anomalies. However, this also limits the recovery of loose clothing. To address this issue, we propose an end-to-end method called IHRPN for reconstructing clothed humans from a single 2D human image. This method includes a feature extraction module for semantic extraction of image features. We propose an image semantic feature extraction aimed at achieving pixel model space consistency and enhancing the robustness of loose clothing. We extract features from the input image to infer and recover the SMPL-X mesh, and then combine it with a normal map to guide the implicit function to reconstruct the complete clothed human. Unlike traditional methods, we use local features for implicit surface regression. Our experimental results show that our IHRPN method performs excellently on the CAPE and AGORA datasets, achieving good performance, and the reconstruction of loose clothing is noticeably more accurate and robust.

## 1. Introduction

In today’s world, technologies such as virtual reality, augmented reality, and mixed reality are becoming increasingly integral to our lives. To cater to these applications, there is a growing need to reconstruct human body models from single images taken in the field [1]. However, this poses a significant challenge as a result of the lack of depth information in a single photo, making it difficult to determine the “thickness of the human body” on the Z-axis.

Despite the considerable advances in graphics card performance, which have led to a surge in deep learning-based research, the reality remains less than ideal. Prior methods have relied heavily on explicit representations like mesh, voxel, depth map, and point cloud [2,3,4]. It was not until the introduction of Pixel-Aligned Implicit Function(PIFu) and High-Definition Pixel-Aligned Implicit Function(PIFuHD) [5,6] that the focus shifted toward implicit representations [7]. Explicit representations can directly depict the geometric shape of the human body, but they come with their own set of drawbacks. They require large amounts of data, leading to substantial memory usage when processing large-scale data, and struggle with complex topological changes such as clothing wrinkles or hair details. On the other hand, implicit representations can handle complex topological changes and generate fine details, but they struggle with maintaining spatiotemporal consistency and can result in disconnected limbs or degraded shapes.

Through the use of scanner-acquired training data and implicit functions for 3D representation, significant progress has been made. However, these methods [8,9] typically employ parametric models as geometric priors, which provide good pose stability but also limit topological flexibility. Some methods recognize clothing types and use specific models for reconstruction, but this approach is difficult to extend to a variety of clothing styles, limiting its generalizability in real-world scenarios.

To address these challenges, particularly the lack of robustness of loose clothing, we first employ a novel feature extraction technique that can understand and process image information at a high semantic level. This means our method can understand and handle more complex features, such as the folds, textures, and colors of clothing, while not losing low-level visual details, such as edges and shapes. Furthermore, our training process continuously challenges the model, enabling it to infer more accurate 3D structures from limited semantic information. This self-challenging process allows our model to better understand and handle various complex visual scenes, including loose clothing.

Next, we use implicit functions for 3D representation, which can provide good pose stability while maintaining topological flexibility. This means our model can effectively handle various complex poses, such as running, jumping, and bending, as well as various complex clothes, such as loose shirts, skirts, and pants. This flexibility allows our model to have good robustness and generalization ability when handling various complex visual scenes.

We propose an implicit 3D human body reconstruction guided by a parameterized model and a normal map, referred to as IHRPN (implicit 3D human reconstruction guided by parametric models and normal maps). Inspired by ICON [8], our method infers the normal map of a clothed human from the input image, continuously optimizes the SMPL-X mesh and normal map during inference using a feedback loop, and finally generates the isosurface using a visibility-aware implicit surface regressor.

The IHRPN is divided into two main parts. The aim of the initial phase is to deduce the SMPL-X mesh from the provided input image. To more effectively capture the features present in the input image, we employ a VQGAN [10] (Vector Quantized Generative Adversarial Network) encoder to transform the original image into discrete semantic tokens. These tokens are then randomly masked, and the resulting masked tokens are mapped onto the parameter space of the SMPL-X, thereby generating the SMPL-X mesh. This process continually challenges the model, enabling it to infer more accurate 3D structures from limited semantic information.

The second part aims to generate a complete clothed human body model based on the SMPL-X mesh and the input image. To generate a more accurate human body model, we render the normal map of the front and back based on the previously generated SMPL-X model using Pytorch3D. We then predict the dressed human body normal map in combination with the input image and, finally, infer the implicit surface (isosurface) through the implicit function.

Our work makes three key technical contributions:

- We proposed VFSM (VQGAN Feature Space Mapper), which is a novel representation learning method in the field of point cloud data. This method excels at capturing intricate features within images and can deduce highly precise 3D structures from a limited set of semantic information.
- In response to the aforementioned innovative aspects, we have introduced a novel loss function. This function aids the network in aligning its output with the ground truth of human posture estimation, thereby facilitating the generation of a more precise SMPL-X mesh.
- We propose IHRPN, a new method for 3D human reconstruction that effectively addresses the robustness issue with loose clothing.

## 2. Related Work

### 2.1. Explicit Representation

The representation of 3D surfaces is a fundamental challenge in the field of 3D learning. Recently, explicit representations such as point clouds, voxel grids, and triangular meshes [11,12,13] have been explored to emulate the success of deep learning techniques applied to 2D images. BodyNet [3] employs intermediate tasks (e.g., segmentation, 2D/3D skeletons) to estimate low-resolution body voxel grids. Volumetric Regression Network (VRN) [14] directly generates a voxel grid from a single image without any intermediate tasks. However, voxel-based methods are typically constrained by low resolution, making it difficult to recover detailed shape information.

Inspired by the efficient processing capabilities of point cloud data and the flexibility of topological modeling, some methods [15,16] try to estimate the coordinates of points from input images. However, to generate meshes containing fine surface details via Poisson reconstruction requires the estimation of a large number of points.

Numerous methods [17,18,19,20] focus on predicting or regressing 3D body meshes from RGB pixels, often overlooking clothing. To incorporate clothed human figures, an alternative approach [21,22] introduces 3D offsets to the body mesh. They are compatible with existing animation pipelines as they inherit the layered skeleton and skin weights of the underlying statistical body model. However, this body + certain offset strategy lacks flexibility. It works well for close-fitting clothes, but it is useless for loose clothes, such as dresses and skirts, whose topology obviously deviates from the body.

To enhance topological flexibility, some methods [23,24] reconstruct 3D clothed human bodies by modeling each category of clothing separately. However, extending this “clothing-independent” approach to accommodate various clothing styles is not straightforward, limiting the model’s generalizability to real-world clothing adaptation. Therefore, developing more flexible and adaptable methods remains an open challenge in the field.

### 2.2. Implicit Representation

The success of deep implicit representations in general object modeling has sparked research interest in 3D human reconstruction [25,26]. Deep implicit functions [7,27] have the ability to represent detailed 3D shapes with arbitrary topologies without any resolution limitations. PIFu [5] introduced pixel-aligned implicit human shape reconstruction, and PIFuHD [6] significantly improved geometric details with a multilevel architecture and normal maps predicted from an RGB image. However, both PIFu and PIFuHD are susceptible to reconstruction artifacts in scenarios of challenging poses and self-occlusions. GeoPIFu [28] introduced a coarse shape of volumetric humans, while Self-Portraits [29], PINA [30], and S3 [31] utilized depth or LiDAR information to regularize the shape and improve the robustness to pose variation. PaMIR [9] integrated implicit functions and parametric body models to propose a novel method of representing 3D human models. However, its generalization capabilities require further improvement in challenging postures and scenarios involving loose clothing. ARCH [32] proposed to regress animation-ready 3D avatars in a canonical pose (A-pose), but it struggles to generate accurate results, especially for loose clothes and human-object interaction, due to the ambiguity in determining the position of objects and accessories in A-pose. imGHUM [33] implicitly models the entire human body as a zero-level set function without using an explicit template network, but the complexity of the computation significantly escalates the demand for computational resources.

## 3. Method

In this section, we focus on the design concept of the method, first introducing the generation of the SMPL-X model from a single image (Section 3.1). We then elaborate on the process of reconstructing a clothed human body model based on SMPL-X (Section 3.2).

### 3.1. Parametric Mesh

In our pursuit to infer a more comprehensive SMPL-X mesh from a single image, we introduce VFSM (VQGAN Feature Space Mapper). SMPL-X employs standard vertex-based linear skinning and learned corrective blend shapes. It comprises N = 10475 vertices and K = 54 joints, covering most body joints, such as the neck, chin, eyeballs, and fingers. VQGAN has the ability to transform images into discrete semantic symbols, thereby aiding in the capture of key image features.

**Figure 1:**
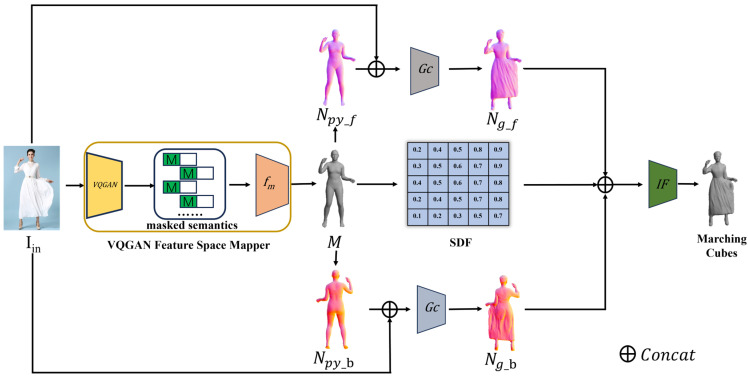
General overview of the IHRPN. The IHRPN method, in two steps, predicts a 3D human body model from a single image. First, it uses the VFSM (VQGAN Feature Space Mapper) to extract image features and predict the SMPL-X mesh. Second, it renders normal maps of the SMPL-X mesh using Pytorch3D, predicts the normal maps of the clothed human body, and generates the final 3D model through an implicit function.

A segmented 2D color image $I_{in}\epsilon R^{3\times 256}$ serves as the input for the network. As shown in Figure 1, VQGAN extracts meaningful human body features $f_{VG}$ from $I_{in}$, such as joint positions, body contours, surface details, etc., and transforms them into discrete semantic symbols $S_{s}$. Subsequently, random masking operations are performed on the semantic symbols to create a scene with missing partial information, challenging the model to infer the three-dimensional structure from limited information. Naturally, it is not feasible to directly feed the masked semantic symbols into the SMPL-X, as the semantic symbols and the parameter space of the SMPL-X mesh are not aligned at the feature level. Therefore, we must transform the features into the same dimension.

To achieve this, we introduce a mapping function, denoted as $f_{m}$. The input to $f_{m}$ consists of discrete semantic symbols obtained after VQGAN masking, which are then processed by a CNN encoder. This is followed by a series of fully connected layers, each succeeded by a ReLU activation function to enhance nonlinearity. The output channels of the final layer correspond to the parameter channels of the SMPL-X model. Upon determining the received parameters, we are now equipped to estimate the SMPL-X model, represented as M(θ,β,φ).

Of course, to urge the model to develop in a more accurate direction, we specially designed the loss function $L_{SMPLX}$ as follows:

$$L_{SMPLX}=\lambda_{pose}\cdot L_{pose}+\lambda_{shape}\cdot L_{shape}+\lambda_{adv}\cdot L_{adv}$$

Among them, $L_{pose}$ is pose loss and $L_{shape}$ is shape loss, which ensure that the generated model’s pose and shape are consistent with the input. Both of them are L2 losses, which are computed by measuring the discrepancy between the generated SMPL-X model and the corresponding ground truth. adv refers to adversarial loss. And λ’s are the weights of various loss terms.

### 3.2. Implicit Human Reconstruction

Given the SMPL-X model, we employ the Pytorch3D differentiable renderer, denoted as $R_{py}$, to render and obtain normal maps $N_{py}=\left\{N_{py\_f},\;N_{py\_b}\right\}$ for both the front and back of the human body. Here, $N_{py\_f}$ represents the normal maps of the front side, i.e., the visible side, while $N_{py\_b}$ signifies the normal maps of the back side, i.e., the invisible side. We then concatenate the input image $I_{in}$ with the normal maps $N_{py}$ and generate the normal maps $N_{g}=\left\{N_{g\_f},\;N_{g\_b}\right\}$ of the clothed human body model through the network $G_{c}$.

$$R_{py}\left(M\right)\to \;N_{py}$$

$$G_{c}\left(N_{py},\;I_{in}\right)\to \;N_{g}$$

The loss function of $G_{c}$ is as follows:

$$L_{G}=L_{N}+\lambda_{VGG}L_{VGG}$$

where $L_{N}=\left|N_{py}-N_{g}\right|$ is the L1 loss between the ground truth and the predicted normal. $L_{VGG}$ is the perceptual loss, and $\lambda_{VGG}$ is the weight.

It is universally acknowledged that the ultimate result of implicit reconstruction is closely linked to the outcomes of human posture estimation. Indeed, one might contend that the caliber of posture estimation establishes the upper limit for the potential success of the implicit reconstruction. Accurate posture estimation acts as a crucial beacon for implicit reconstruction, while inadequate estimation can lead the reconstruction process astray. Consequently, to improve posture estimation precision, we exploited the disparity between the normal map rendered by SMPL-X and the predicted normal map of the clothed human body during the inference process to optimize the SMPL-X fits.

Upon securing the iteratively optimized normal maps $N_{g}$ of the clothed human figure and the SMPL-X mesh M, we are now in a position to infer the final implicit surface. We extract three dimensions from the normal maps of the clothed human body, another three dimensions from the M, and an additional dimension from the Signed Distance Function (SDF), culminating in a total of seven dimensions. These dimensions are then fed into the implicit function F, generating implicit surfaces based on local feature regression.

$$IF=\left\{\begin{array}{c} occupied,\;\;\;s>0.5\\ on\;the\;surface,\;s=0.5\\ unoccupied,\;\;s<0.5 \end{array}\right.$$

Consider a point, denoted as P. Our objective is to ascertain whether it is occupied by an object. To achieve this, we define an “occupancy probability”, symbolized as “s”, which indicates the probability that point P is occupied by an object. If “s” surpasses 0.5, we infer that point P is indeed occupied by the object. On the other hand, if “s” is less than 0.5, we deduce that point P is not occupied by the object. In instances where “s” is equal to 0.5, we interpret this as point P being located on the surface of the object. Finally, we employ the Marching Cube algorithm [34] to extract the isosurface, resulting in the final model of human body reconstruction.

## 4. Experiments and Results

### 4.1. Datasets

In this study, our main comparison is between our method and those of PIFu, PIFuHD, and ICON. We use the Thuman2.0 dataset [35] for training, which is a specialized dataset focusing on 3D human body models. The Thuman2.0 dataset, obtained through the DSLR (Digital Single-Lens Reflex) collection, captures intricate details of the human body, such as wrinkles and skin texture. It comprises 526 high-quality 3D human body scans and holds significant application value in fields such as 3D human body reconstruction, animation, virtual reality (VR), augmented reality (AR), and game development.

We perform both quantitative and qualitative evaluations on the CAPE [21] and AGORA [36] datasets. The CAPE dataset aims to provide a comprehensive and diverse collection of 3D human body models that include different body shapes, postures, and various clothing styles. It employs a parametric representation method to generate a wide array of realistic 3D human samples by adjusting the shape, posture, and clothing parameters of the human model. The AGORA dataset, known for its realism and accuracy, is used for training and evaluating the estimation of 3D human posture from images. It includes human body models and a substantial number of synthetic images depicting various postures, body shapes, and clothing. Each image is equipped with extensive annotation information, including 3D/2D human keypoints, 3D human model parameters, 2D human segmentation masks, and more.

### 4.2. Metrics

**Point-to-surface distance (cm)**: The point-to-surface distance measures the distance between the point cloud data in the reconstructed model and the actual surface. A smaller point-to-surface distance means that the reconstruction result is closer to the actual surface and that the reconstruction accuracy is higher.

**Chamfer distance (cm)**: The Chamfer distance is a symmetrical distance measure. For each point in the reconstructed model, find the point on the actual surface closest to this point and calculate the distance; similarly, for each point on the actual surface, also find the point in the reconstructed model closest to this point and calculate the distance. The average of these two groups of distances is the Chamfer distance. A smaller Chamfer distance indicates that the two groups of point clouds are closer and the reconstruction effect is better. 

**Normal difference (L2)**: The normal difference is used to evaluate the consistency between the normal direction of the reconstructed surface and the normal direction of the actual surface. This indicator calculates the angle between the normal of the nearest point on the actual surface and the reconstructed normal. A smaller normal reprojection error means that the normal direction of the reconstructed surface is more consistent with the actual surface, and the reconstructed geometric shape is more accurate.

### 4.3. Implementation Details

We train on 526 models from the THuman2.0 dataset and select 50 models each from the CAPE and AGORA datasets for testing. We use VFSM to extract discrete semantic features from 512 × 512 input images. The encoder consists of five blocks, each containing two residual blocks. After each block in the encoder, we use average pooling for feature downsampling, reducing the size by half. After adjusting the feature dimensions through a mapping function, we use a codebook with 1024 entries to quantize each pixel of the encoder’s output feature map. Each entry in the codebook has a dimension of 256. The decoder consists of another five blocks, each containing two residual blocks. After each block in the decoder, the feature map is upsampled to double its original size. Finally, we output 119 parameters to generate the SMPL-X model. We use the ADAM optimizer with a learning rate of 1.0 × 10^−4^, which converges in 75 epochs.

### 4.4. Evaluation

**Quantitative evaluation**. As illustrated in Table 1, we carry out a comparative study between IHRPN and other established methods, namely PIFu, PIFuHD, and ICON. To maintain a level playing field, all methods are trained on the Thuman2.0 dataset, ensuring a standardized input across all methods. Simultaneously, we perform a quantitative analysis on two additional datasets, CAPE and AGORA, to more comprehensively evaluate the generalization capabilities. A clear observation from rows 3–6 in Table 1 reveals that IHRPN outperforms the other three methods. Although ICON’s performance is comparable to that of IHRPN, it lags due to inadequate image feature extraction. PIFu and PIFuHD, lacking a prior shape, are prone to generating irrelevant limbs or limbs detached from the body, thereby leading to the most significant errors. In summary, IHRPN has achieved state-of-the-art (SOTA) performance across both datasets.

**Network complexity**. The complexity of the networks is reported in column 8 of Table 1. Given the five methods, each method tests the time to generate a 3D human model from human images, with each method testing 50 images and taking the average. As can be seen from column 8 of the table, PIFuHD takes the longest time because it adopts a coarse-to-fine, two-stage processing process. Next is PIFu, which uses a pixel-aligned implicit function to reconstruct the human body, eliminating the need for PIFuHD’s multi-level processing. ICON recovers 3D shapes from normal information, making it more direct and efficient in computation. Our method is faster than the above three methods due to more efficient image feature extraction. In the absence of VFSM, we use CNN to replace the feature extraction module, so its overhead is the shortest.

**Figure 2:**
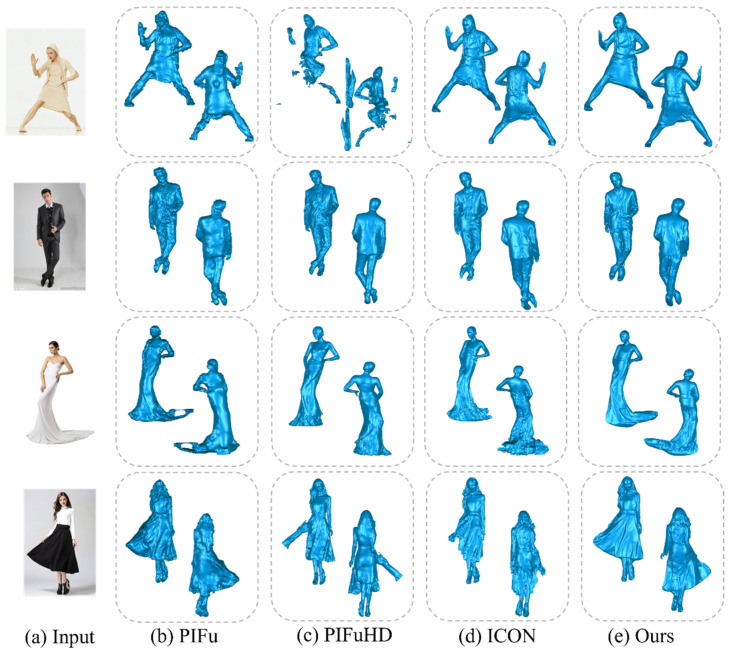
Qualitative evaluation of (**a**) the input image, (**b**) the experimental results of PIFu, (**c**) the experimental results of PIFuHD, (**d**) the experimental findings of ICON, and (**e**) the results obtained through our method.

**Qualitative evaluation**. As shown in Figure 2, our method has clear advantages compared to the other three methods. ICON struggles to generate appropriate topology when dealing with loose clothing. PIFu requires a large spatial context and has a lower-resolution input image, resulting in less detailed 3D estimates. PIFuHD is influenced by the input image and the predicted normal maps on both sides, making the model more sensitive to background information. Our method demonstrates a significant advantage in handling loose clothing. It reliably generates an appropriate topology, unlike other approaches. Moreover, it exhibits robustness for loose garments. This means our model can accurately estimate 3D shapes across different clothing types and human poses.

### 4.5. Ablation Study

We set up an ablation study specifically for the feature extraction module to monitor the presence of VFSM and its impact on model performance. The results are shown in row 7 of Table 1. In the absence of VFSM (i.e., without the image feature extraction module), we use CNN for feature extraction to generate the SMPL-X model. Compared to row 3, when using CNN for feature extraction, all metrics significantly increase, far exceeding the scenario when using VFSM.

**Figure 3:**
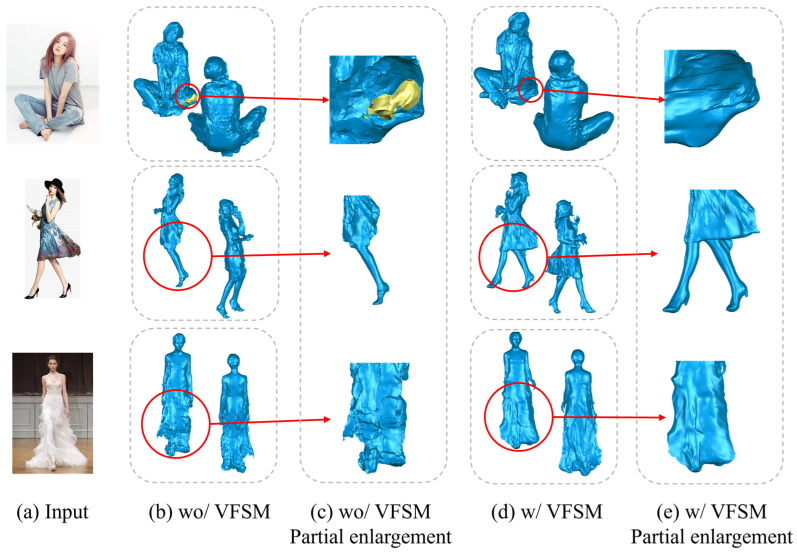
Ablation study. (**a**) is the input image, (**b**) shows the results without VFSM, (**c**) is a magnified local view, (**d**) demonstrates the experimental results with VFSM, and (**e**) is similar to (**c**), providing another local magnified view.

In Figure 3, it can be seen that when our feature extractor is not used, the reconstruction results are full of flaws. The first row of column (c) even shows holes (the yellow part in the figure represents holes), the legs in the second row of column (c) are distinctly different from the figure, and the back of the skirt is not fully modeled. The third row of column (c) shows that the lower half of the model is flat and lacks depth, and the shape of the legs is not discernible.

In comparison, the results displayed in column (e) exhibit substantial enhancement. It is worth noting that the reconstruction output has an almost perfect structure, with virtually no gaps visible to the naked eye. Moreover, it displays a seamlessly aligned human structure that corresponds precisely with the input image. This underscores the immense advantages offered by our feature extractor in facilitating SMPL-X inference. This shows that the feature extraction module we have introduced proves to be pivotal in the realm of 3D human body reconstruction.

## 5. Discussion

We introduce a novel image feature extraction module that can transform the features of the input image into semantic attributes. This allows IHRPN to understand and handle more complex features, such as the folds and textures of clothing, while retaining low-level visual details. Our method cleverly handles complex visual scenes, inferring accurate 3D structures from limited information. Our experiments show that the model can accurately reconstruct loose clothing, greatly improving the robustness to loose clothing. However, the reconstruction quality of IHRPN is not only affected by the feature extraction module but also by the accuracy of the predicted normal map of the clothed human body. Inaccurate prediction of the normal map may lead to inconsistencies in the posture of the reconstructed model and reduce the robustness of different postures and types of clothing. In the future, we will focus on improving the accuracy of the predicted normal map to enhance the precision of the reconstructed model.

## 6. Conclusions

In this paper, we introduce IHRPN, a novel method for reconstructing 3D clothed human bodies from a single image. This innovative approach is designed to tackle the long-standing challenge in the field of 3D human body reconstruction—dealing with loose clothing. To enhance the robustness against loose clothing, we explored an image feature extraction module based on VQGAN, known as VFSM. This module significantly improves the efficiency and accuracy of feature extraction and transformation, enabling our method to handle complex clothing textures and patterns more effectively. IHRPN stands out due to its superior performance on three evaluation metrics, demonstrating its effectiveness and robustness in various scenarios. The state-of-the-art (SOTA) results achieved by our method attest to its efficacy. These results not only validate the effectiveness of our approach but also set a new benchmark in the field of 3D human body reconstruction.

## Figures and Tables

**Table 1:** Quantitative evaluation and ablation experiments on the CAPE and AGORA datasets. “wo/VFSM” refers to the ablation experiment conducted without VFSM. “Execution Time” denotes the time required by each method to reconstruct a 3D human body model from a single image. ↓ and bold represent that lower values are better for the corresponding indicators and highlighting the optimal performance among the various metrics respectively.

Dataset	CAPE			AGORA			Execution Time ↓
Method	Chamfer ↓	P2S ↓	Normals ↓	Chamfer ↓	P2S ↓	Normals ↓	
**Ours**	**1.163**	**1.296**	**0.059**	**1.202**	**1.586**	**0.061**	20.1 s
PIFu	3.587	3.652	0.119	3.379	3.452	0.092	33.7 s
PIFuHD	3.103	2.897	0.113	3.087	3.326	0.084	41.6 s
ICON	1.187	1.358	0.065	1.221	1.593	0.063	21.4 s
wo/VFSM	2.039	1.414	0.078	1.974	1.651	0.072	**19.6 s**

## Data Availability

Data underlying the results presented in this paper are not publicly available at this time but may be obtained from the authors upon request.
